# Longer than 2 hours to antibiotics is associated with doubling of mortality in a multinational community-acquired bacterial meningitis cohort

**DOI:** 10.1038/s41598-021-04349-7

**Published:** 2022-01-13

**Authors:** Damon P. Eisen, Elizabeth Hamilton, Jacob Bodilsen, Rasmus Køster-Rasmussen, Alexander J. Stockdale, James Miner, Henrik Nielsen, Olga Dzupova, Varun Sethi, Rachel K. Copson, Miriam Harings, Oyelola A. Adegboye

**Affiliations:** 1 The Townsville University Hospital, Angus Smith Drive, Douglas, QLD 4814 Australia; 2 College of Medicine and Dentistry, James Cook University, Discovery Drive, Douglas, QLD 4814 Australia; 3 Department of Infectious Diseases, Aalborg University Hospital, Mølleparkvej 4, 9000 Aalborg, Denmark; 4 The Research Unit for General Practice, Department of Public Health, University of Copenhagen, 1014 Copenhagen K, Denmark; 5 Institute of Infection and Global Health, University of Liverpool, Liverpool, L69 7BE UK; 6 Hennepin County Medical Center, University of Minnesota, Minneapolis, USA; 7 Third Faculty of Medicine, Charles University, and University Hospital Bulovka, Prague, Czech Republic; 8 Public Health and Tropica Medicine, College of Public Health, Medical and Veterinary Sciences, James Cook University, 1 James Cook Drive, Douglas, QLD 4814 Australia; 9 Australian Institute of Tropical Health and Medicine, Discovery Drive, James Cook University, Douglas, QLD 4814 Australia

**Keywords:** Infection, Meningitis, Statistics

## Abstract

To optimally define the association between time to effective antibiotic therapy and clinical outcomes in adult community-acquired bacterial meningitis. A systematic review of the literature describing the association between time to antibiotics and death or neurological impairment due to adult community-acquired bacterial meningitis was performed. A retrospective cohort, multivariable and propensity-score based analyses were performed using individual patient clinical data from Australian, Danish and United Kingdom studies. Heterogeneity of published observational study designs precluded meta-analysis of aggregate data (I^2^ = 90.1%, 95% CI 71.9–98.3%). Individual patient data on 659 subjects were made available for analysis. Multivariable analysis was performed on 180–362 propensity-score matched data. The risk of death (adjusted odds ratio, aOR) associated with treatment after two hours was 2.29 (95% CI 1.28–4.09) and increased substantially thereafter. Similarly, time to antibiotics of greater than three hours was associated with an increase in the occurrence of neurological impairment (aOR 1.79, 95% CI 1.03–3.14). Among patients with community-acquired bacterial meningitis, odds of mortality increase markedly when antibiotics are given later than two hours after presentation to the hospital.

## Introduction

Community-acquired bacterial meningitis (CABM) is a deadly condition of global significance^1^. Despite effective vaccinations against the main CABM pathogens, the disease remains frequently fatal and appropriate antibiotic therapy with adjuvant corticosteroids are lifesaving. As with treatment for sepsis, evidence suggests that the earliest institution of anti-bacterials is associated with optimal clinical outcomes^2,3^. However, the relationship between time to antibiotics (TTA) and death or neurological impairment has not been fully elucidated. Treatment recommendations for CABM^4^ are unable to rely on randomised clinical trial data to guide the minimum time to effective therapy. Practice guidelines for the treatment of bacterial meningitis conclude that there are inadequate data to specify the optimal time between clinical assessment and the institution of antimicrobial therapy^5^.

It is worthwhile, therefore, to utilise all the available data on CABM outcomes in relation to TTA to attempt to be as precise as possible in prediction of clinical outcomes after treatment of this condition. We conducted a systematic review of the medical literature to identify papers that describing the association between TTA and clinical outcomes in adults with CABM. We attempted meta-analysis using the reported aggregate data. An individual patient data meta-analysis was performed using data from four separate hospital practice settings in Australia (The Townsville University Hospital, this paper), Denmark (two non-overlapping studies)^6,7^, and the United Kingdom (UK)^8^. Our primary aim was to determine the association between TTA and death due to CABM during the hospital admission for this disease. The secondary aim was to analyse the association between TTA and neurological impairment due to CABM. We used propensity-matched individual patient data to attempt to adjust for confounding factors and optimally estimate the association between TTA and death and neurological impairment in patients with CABM using available retrospective cohort information.

## Methods

### Systematic review of publications describing time to antibiotics in treatment of CABM

A systematic search of Medline/PUBMED and EMBASE (both 1947–present) databases according to PRISMA guidelines^9^ was undertaken to identify publications describing a relationship between TTA and clinical outcomes in CABM (Figure S1). Search terms included keywords and medical subject heading terms (MESH) ‘bacterial meningitis’, ‘community-acquired bacterial meningitis’, ‘anti-bacterial agents’, ‘antibiotics’ and ‘time’ (See Supplementary Table S1 for search strategy). The search had no restriction on language. A search of the grey literature, as well as reference lists of key articles, was undertaken.

Articles were independently evaluated by title, abstract and full-text by two authors (EH and MH). Articles selected were reviewed by a third author (DE), who also resolved any disagreement over suitability for study inclusion. Eligible studies were those that reported time to antibiotic administration and our primary outcome, death due to CABM and secondary outcome of neurological impairment in adults with CABM.

### Cohort of adult patients with CABM from the Townsville University Hospital (TUH)

Clinical data relating to TUH patients with International Classification of Disease Edition 10 Australian Modification (ICD-10-AM) discharge codes for bacterial meningitis (see Supplementary Table S2) during the period 1/1/2006 and 30/6/2018 were retrieved from medical records. The case definition of proven CABM used for TUH cases was in keeping with other studies where individual data were made available and required the presence of: clinical syndrome including at least one of headache, meningism, photophobia, or fever together with, microbiological proof of bacterial meningitis as shown by positive cerebrospinal fluid (CSF) or blood cultures (in the presence of CSF pleocytosis) or polymerase chain reaction (PCR) for pathogens associated with CABM.

Data relating to host demographics, comorbidities, clinical characteristics, antibiotic therapy, and clinical outcomes were collected. TTA was recorded from hospital admission notes including from the Emergency Department. Appropriate antibiotic therapy was defined as any intravenous antibiotic administered in doses capable of penetrating the blood–brain barrier to which the meningitis pathogen was susceptible. The Glasgow Outcome Scale (GOS), a validated scale ranging from one (dead) to five (good recovery with return to original functional level), was used to determine neurological impairment. A score of four or less corresponds to some degree of neurological impairment, with a score of four described as ‘moderate disability–disabled but independent’^10^.

Ethical approval for the use of these data was granted Townsville Hospital and Health Service (THHS) Human Research Ethics Committee (HREC18/QTHS/140). All methods were performed in accordance with the relevant guidelines and regulations/Declaration of Helsinki. A waiver of consent for access to TUH patient data was approved under the Queensland Public Health Act (RD007478).

### Individual patient data collection

In an attempt to combine all individual patient data from the identified publications, corresponding and senior authors were contacted by email on at least three occasions to seek their involvement in this study.

### Statistical analysis

Descriptive data were reported in standard formats. Binary outcome variables were analysed using univariate and multivariable logistic regression. Stepwise selection (backward and forward) was used to select important predictors of health outcomes from the full model.

To account for unobserved inter-study variability among patients, clinical outcomes within studies were treated as correlated random effects. Generalizsed Linear Mixed Models were constructed for binary outcome variables (multivariable mixed logistic regression) and were used to estimate the association between time to treatment (antibiotic therapy) in hours and mortality (GOS = 1) and neurological impairment (GOS = 2–4), separately adjusting for other covariates. The associations were presented as adjusted odds ratio (aOR) for outcomes associated with time to antibiotics and selected covariates.

Propensity scoring was used to select a cohort of CABM patients matched for age and gender that had the same logistic probability of receiving treatment within a specific time. Exact matching for gender and age was undertaken utilising one-to-one matching using nearest neighbour matching within a calliper of 0.25.

Statistical analysis was conducted in SAS 9.4 (SAS Inc. Cary NC) and R software version 3.4.2^11^.

## Results

Database searches found 778 publications matching search terms. Following removal of duplicates, title and abstract screening and full-text review, 18 publications were suitable for qualitative analysis (see Table 1 and Figure S1 PRISMA^9^ diagram). Of these, eight studies reported effect estimates of CABM outcomes^6,12–18^ in relation to time to antibiotics and were considered for possible meta-analysis. Seven of these eight studies showed an association between increasing TTA and poor outcomes.

**Table 1:** Characteristics of studies of community-acquired bacterial meningitis that include time to antibiotic data.

Study	Site	Design	N^a^	Setting	Age^b^	Main bacterial pathogens^c^	Outcomes measured	Mortality (%)	Risks of delay in antibiotics (95% confidence intervals)^d^
**Studies reporting risk associated with time to antibiotics**									
Bodilsen 2016^18^	Denmark 1998–2014	Regional retrospective	173	General	58	*S. pneumoniae 55%, N. meningitidis 21%,* other bacteria 24%	In-hospital mortality and neurological impairment	23.3	Mortality TTA > 6 h RR 1.6 (0.8–3.2)
Køster-Rasmussen 2008^6^	Denmark 2002–2004	Regional retrospective	125	General	62	*S. pneumoniae 62%, N. meningitidis 9%, Listeria spp. 6%, S. aureus 5%*	Death or neurological impairment at follow-up (median 30 days)	33	Poor outcome TTA OR/h 1.09 (1.01–1.19)
Auburtin 2006^11^	France 2001–2003	Multicentre prospective	156	ICU	56	*S. pneumoniae* 100%	Mortality and neurological impairment at ICU admission and 3 months	33	Mortality TTA > 3 h OR 14.12 (3.93–50.9)
Bretonniere 2015^12^	France 2004–2008	Multicentre retrospective	157	ICU	41	*S. pneumoniae 56%, N. meningitidis 32%*	ICU mortality, 3 month mortality and neurological impairment	15	Median TTA in survivors 1·6 h/3.8 non-survivors (p = 0.003)
Dzupova 2009^13^	Czech Republic 1997–2006	Single centre prospective	279	General	51	*S. pneumoniae 29%, N. meningitidis 27% , L. monocytogenes 7%,* Enterobacteriaceae 4%	Mortality and neurological impairment at 6 months	20	Unfavourable outcome TTA > 48 h post CABM symptoms OR 2.47 (1.04–5.88)
Glimaker 2015^14^	Sweden 2005–2012	National registry retrospective	712	General	61	S*. pneumoniae 51%, N. meningitidis 12%, H. influenzae 7%,* Streptococcus spp. 6%	In-hospital mortality, neurological impairment at 2–6 months post-discharge	10	Mortality TTA RR/h 1.13 (1.03–1.23)
Lepur 2007^15^	Croatia 1990–2004	Single centre retrospective	286	General	49	*S. pneumoniae 39%, N. meningitidis 8%, L. monocytogenes 7%, H. influenzae 6%*	In-hospital mortality, neurological impairment at discharge	22.7	Unfavourable outcome TTA > 24 h OR 2·8 (1·13–7)
Proulx 2005^16^	Canada 1990 –2002	Single centre retrospective	123	General	54	*S. pneumoniae 56%, S. aureus 9%, N. meningitidis 9%, S. aureus 9%, L. monocytogenes 9%*	In-hospital mortality	13	Mortality TTA > 6 h OR 8.4 (1.7–40.9)
**Studies without calculated risk associated with time to antibiotics**									
Abulhasan 2013^17^	Canada 2000–2010	Single centre retrospective	37	ICU	37	*S. pneumoniae* 38%, Group A streptococci 11%, *E. coli 11%, N. meningitidis 8%*	Mortality and neurological impairment at 1 and 3 months		
Aronin 1998^18^	USA 1970–1995	Multicentre prospective	269	General	57	*S. pneumoniae 48%, N. meningitidis 15%, S. aureus* 9%, other streptococci 12%	In-hospital death and neurologic impairment at discharge		
Bryan 1986^19^	USA 1979–1984	Single centre retrospective	14	General	Not reported	*S. pneumoniae 64%, N. meningitis 14%*	Mortality in hospital, neurologic impairment at time of discharge		
Fang 2000^20^	Taiwan 1986 –1999	Single centre retrospective	18	General	51	*Klebsiella pneumoniae 100%*	In-hospital mortality, neurological impairment at discharge		
Lazzarini 2008^21^	Italy 2002–2005	National prospective	322	General	54	*S. pneumoniae 41%, N. meningitidis* 14%, CSF Gram stain positive 41%	In-hospital mortality, neurological impairment at discharge		
Lu 2002^22^	Taiwan 1986–1999	Single centre retrospective	109	General	50	*K. pneumoniae 40%, S. pneumoniae* 9%, viridans streptococci 10%, *S. aureus 6%*	In-hospital mortality		
Milhaud 1996^23^	France	Single centre prospective	39	ICU	67	*S. pneumoniae 36%, L. monocytogenes 23%, S. aureus 18%, K. pneumoniae 5%*	In-hospital mortality		
Miner 2001^24^	USA 1987 –1997	Single centre retrospective	44	General	47	*S. pneumoniae 41%, N. meningitidis 16%, S. aureus 14%, E. coli 9%*	In-hospital mortality		
Mishal 2008^25^	Israel 1988–1999	Single centre retrospective	25	General	Not reported	*S. pneumoniae 28%, N. meningitidis 28%*	In-hospital mortality, neurological impairment at discharge		
Stockdale 2011^8^	UK 2005–2010	Single centre retrospective	39	General	39	*N. meningitidis 51%, S. pneumoniae 44%*	In-hospital mortality		
**Updated data from Bodilsen 2016**^18^									
Bodilsen 2018^7^^e^	Denmark 1998–2014	Regional retrospective	358	General	56.7	*S. pneumoniae 54*·3*%, N. meningitidis 21%,* other bacteria 24·7%	In-hospital mortality and neurological impairment	23.5	Mortality TTA > 4 h (32%) vs. < 2 h (15%), p = 0.003

*CABM* community-acquired bacterial meningitis, *TTA* time to antibiotics, *ICU* intensive care unit, *UK* United Kingdom, *US* United States.

^a^Number of adult patients with CABM.

^b^Mean / median variously reported.

^c^Pathogens with incidence ≥ 5%.

^d^Multivariate model.

^e^The primary outcome of this study was associated with the timeliness of CABM diagnosis. Time to antibiotics, age, type of bacterial pathogen, in-hospital mortality and neurological impairment were among the variables collected.

Heterogeneity of published observational study designs precluded meta-analysis of aggregate data (I^2^ = 90.1%, 95% CI 71.9–98.3%). This was due to marked differences in reporting of time function between admission and antibiotic administration and reported measures of clinical outcome association as described in Table 1. Additionally, interval to the measurement of mortality and neurological impairment, methods of data analysis, and predictive co-variables differed across the studies. A further ten studies^8,19–27^ that described data on TTA but did not calculate associated outcomes were suitable for individual patient analysis.

### The Townsville University Hospital CABM cases

Eighty-three patients from the period 1/1/2006 to 30/6/2018 with the specified ICD-10-AM codes for bacterial meningitis were identified. Twenty-five of these patients met the inclusion criteria. Patient characteristics, aetiological agents, time to effective antibiotics, and clinical outcomes are listed in Table 2 together with data made available by other authors for the individual patient outcome analysis.

**Table 2:** Description of all patient data available for study.

Characteristics	Studies					Overall
	TUH	Bodilsen 2016^7^	Køster-Rasmussen 2008^6^	Stockdale 2011^8^	Miner (unpublished data)	
Number· of patients	25	358	132	39	106	659
Male to female	16:09	179:179	58:74	21:17	NA	274:279
Age, years (mean ± SD)	36.2 ± 22.0	56.7 ± 18.4	57.1 ± 19.5	44.4 ± 20.7	26.3 ± 28.0	50.8 ± 23.5
Time to antibiotics, hours (mean ± SD)	0.9 ± 2.0	9.9 ± 19.8	9.6 ± 34.5‡	2.6 ± 3.7	5.1 ± 9.1	8.3 ± 21.7
Case fatality rate (%)	8	23.5	30.3	18.4	8.5	21.6
Neurological deficit (%)	10.5	29.9	58.6	NR	20.8	32.4
**Infecting bacteria (n, %)**						
*S. pneumoniae*	7 (28)	194 (54.3)	77 (58.8)	17 (45)	NR	295 (54.3)
*N. meningitidis*	13 (52)	58 (16.2)	10 (13.7)	20 (53)	NR	101 (15.7)
*S. aureus*	0	21 (5.9)	6 (4.6)	0	NR	27 (4.2)
*H. influenzae*	2 (8)	16 (4.5)	2 (1.5)	0	NR	20 (3.7)
Others^a^	2 (8)	52 (14.6)	22 (16.8)	1 (2)	NR	77 (14.2)
Other Gram negative^b^	1 (4)	17 (4.7)	6 (4.5)	0	NR	24 (3.7)

^a^Includes *Streptococcus spp·, E. faecalis, L. monocytogenes.*

^b^Includes *E. coli, K. pneumoniae, P. multocida, Capnocytophaga spp*.

*TUH* The Townsville University Hospital, *SD* standard deviation, *N* number, *NR* not reported.

Individual patient data were shared from previous studies by Koster-Rasmussen et al.^6^ and Stockdale et al.^8^. Dr Bodilsen provided updated data^7^ from a recent study, Bodilsen et al.^18^, which was not part of our systematic review. Individual patient data were also made available from another study^14^ where patients were treated immediately on arrival at a tertiary hospital after a variable time to CABM diagnosis at referring hospitals. The time from referring hospital presentation to antibiotic administration were not recorded, so these individual patient data could not be used. Institutional review board approval had been granted for the collection of all these data^6–8,18,26^. Other authors attempted to share individual patient data but were unable to retrieve these due to them being stored on redundant media^17,26^. The remaining authors did not reply to repeated invitations or declined to share data.

Data describing 659 individual patients were available for analysis from the three previously reported hospital-based cohorts^6–8^ and the newly described TUH CABM cases. Unpublished data, were provided by Dr Miner (personal communication) as that included only patient age, time to antibiotics and outcomes. These data were collected with IRB approval, as a pilot study for a later publication^26^.

**Figure 1:**
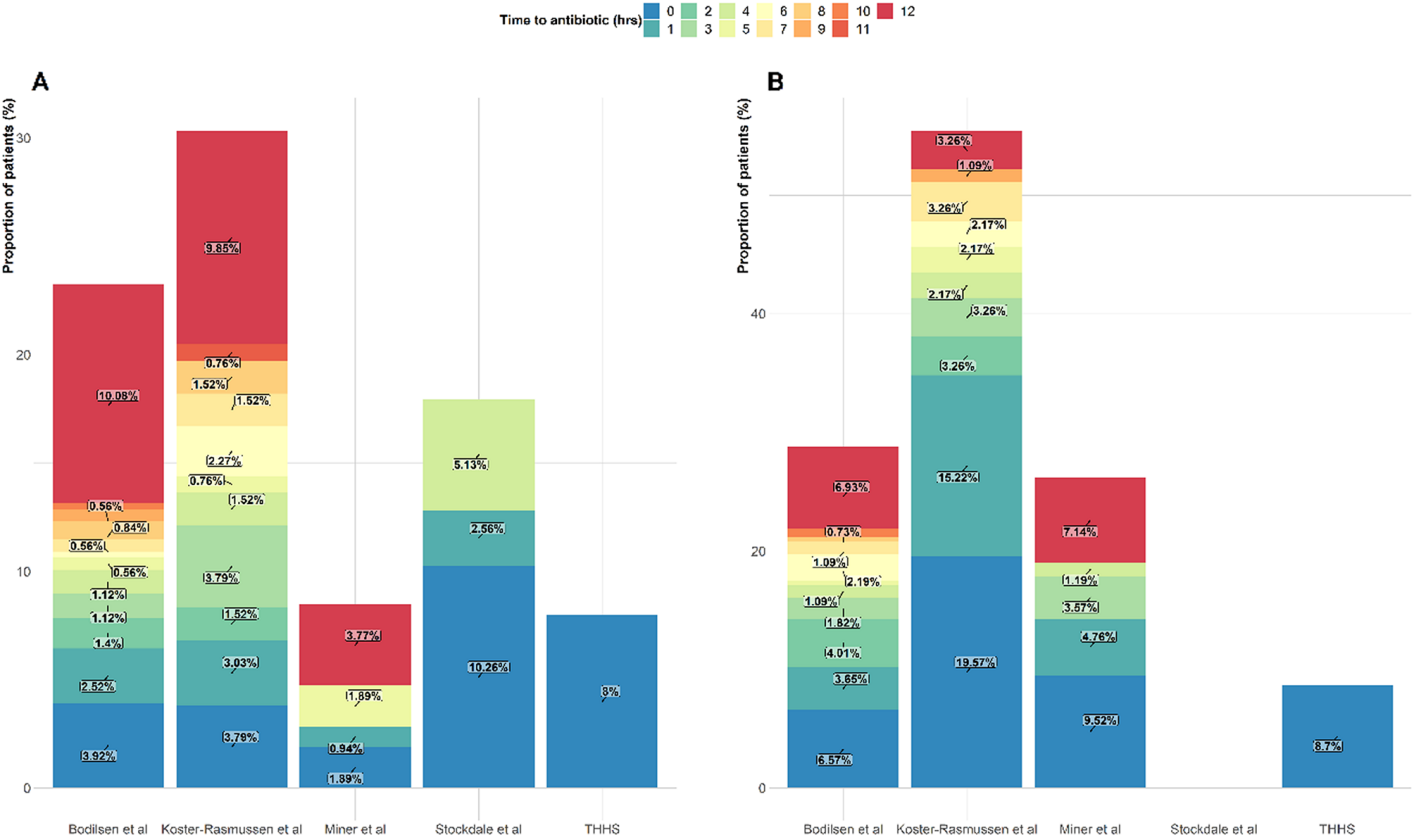
Plot showing proportion of patient with an outcome in relation to time to antibiotics in patients with community-acquired bacterial meningitis across and within each study. (**A**) mortality and (**B**) neurological impairment.

Demographic, clinical, microbiological characteristics, TTA as well as outcomes among studies providing individual patient data available are summarised in Table 2 and Fig. 1. In-hospital death and neurological impairment were described in TUH data and by Bodilsen et al.^7^ Death and neurological impairment due to CABM were measured at 30 days. Stockland et al.^8^ only reported death and not neurological impairment due to CABM. Outcomes due to CABM in the combined dataset were: death rate (21.6%, 95% CI 18.9–24.2) and frequency of neurological impairment (32.4%, 95% CI 30.0–36.0). A total of 563/659 (85.4%) patients received antibiotic therapy within the first 12 h of admission with a mortality of 103/563 (18.3%). Among patients with TTA > 12 h mortality was 39/96 (40.6%).

The most common organisms among the CABM patients studied were *Streptococcus pneumoniae* and *Neisseria meningitidis*. In both the Danish studies^6,7^ a considerable proportion of CABM (14.6%^7^ and 16.8%^6^) were due to streptococcal species, *Enterococcus faecalis,* and *Listeria monocytogenes*. A further group of infrequent bacterial causes of CABM (*Escherichia coli, Klebsiella pneumonia, Pasteurella multocida,* and *Capnocytophaga spp*.) are referred to as ‘other Gram-negatives’.

**Figure 2:**
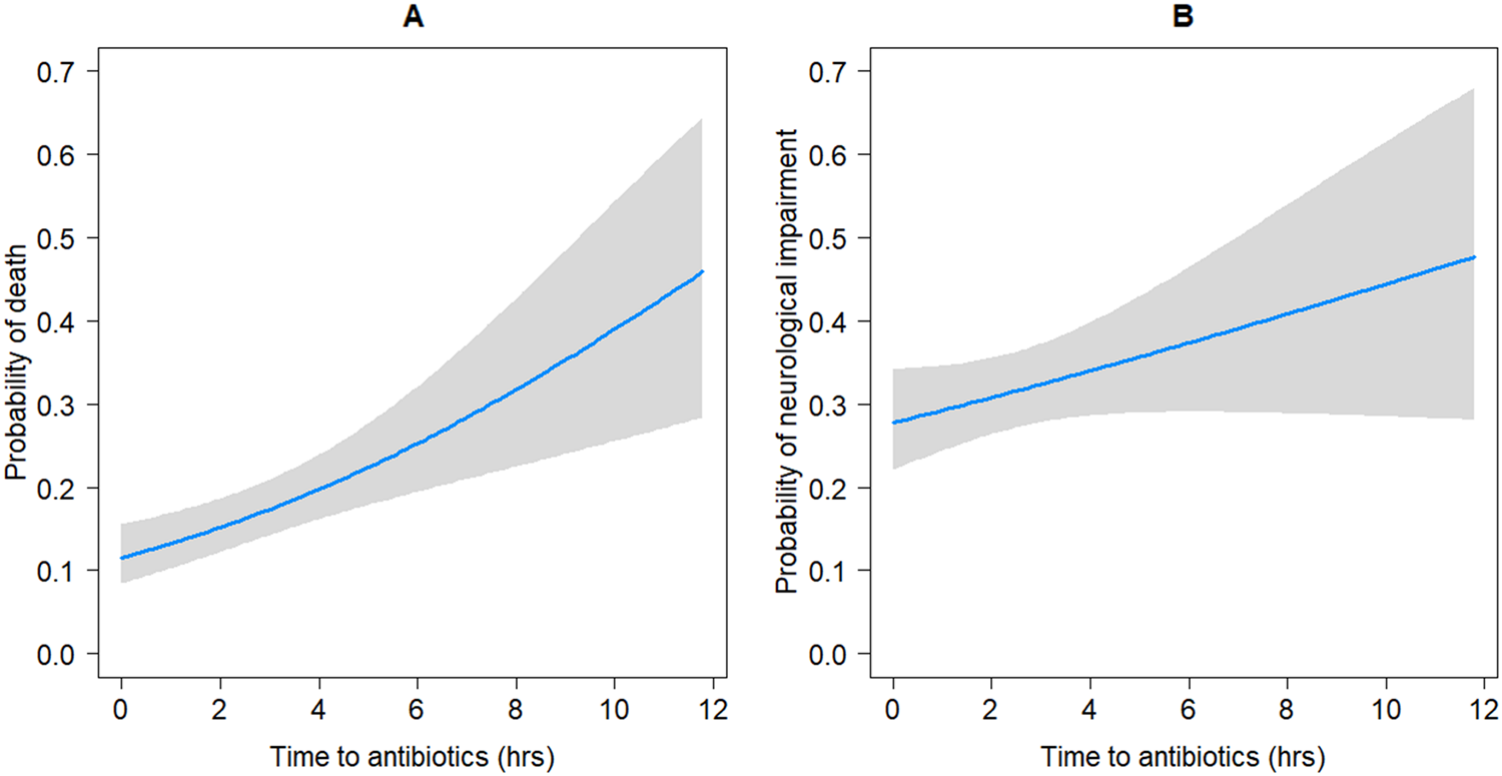
Probability of observing (**A**) mortality and (**B**) neurological impairment in community-acquired bacterial meningitis as a function of time to antibiotic therapy.

Analysis of the combined CABM data showed that the probability of mortality or neurological impairment was associated with increasing TTA (Table 3 and Fig. 2). In this cohort of patients, the probability of death following hospital admission was 11.5% (95% CI 8.0–15.1%) and the probability of neurological impairment was 27.8% (95% CI 21.7–33.8%) (Fig. 2). After a delay of 12 h to appropriate anti-bacterial therapy the probability of both death and neurological impairment were 46.7% (95% CI 27.4–66.1%) and 48.1% (95% CI 26.4–69.6%), respectively. By univariate analysis, increased TTA was associated with increased mortality (OR 1.8 , 95% CI 1.1–2.9) in patients treated after one hour compared to (OR 3.0. 95% CI 2.0–4.5) patients treated after five hours (Table 3).

**Table 3:** Factors associated with mortality and neurological impairment due to community-acquired bacterial meningitis including time to antibiotics by logistic regression.

	Mortality					Neurological impairment				
	Total	Alive	Dead	Odds ratio (95% CI)		Total	Alive	Dead	Odds ratio (95% CI)	
				Univariate	Multivariable				Univariate	Multivariable
Number of patients^a^	659 (100%)	517 (78.6%)	142 (21.6%)		n = 466	485 (100%)	328 (67.6%)	157 (32.4%)		n = 353
Time to antibiotics, h^b,c^	2.0 (0.9–6.0)	1.8 (0.8–4.5)	4.5 (1.5–18.0)	1.0 (1.0–1.1)	1·1 (1·0–1·1)	1.8 (0.9–4.9)	1.8 (0.8–4.2)	2.3 (1.0–6.8)	1.0 (1.0–1.1)	1.0 (1.0–1.1)
> 1 h (vs < 1 h)^a^	461 (74.1)	356 (77.4)	105 (22.6)	1.8 (1.1–2.9)		337 (73.1)	220 (65.3)	117 (34.7)	1.4 (0.9–2.1)	
> 2 h (vs < 2 h)^a^	328 (52.7)	236 (72.2)	92 (27.8)	2.8 (1.8–4.2)		226 (49.0)	146 (64.6)	80 (35.4)	1.2 (0.8–1.8)	
> 3 h (vs < 3 h)^a^	258 (41.5)	177 (68.9)	81 (31.1)	3.1 (2.0–4.6)		172 (37.3)	105 (61.1)	67 (38.9)	1.5 (1.0–2.3)	
> 4 h (vs < 4 h)^a^	214 (34.4)	142 (66.7)	72 (33.4)	3.1 (2.1–4.7)		140 (30.4)	84 (60.0)	56 (40.0)	1.6 (1.0–2.4)	
> 5 h (vs < 5 h)^a^	178 (28.6)	116 (65.5)	62 (34.8)	3.0 (2.0–4.5)		115 (25.0)	67 (58.3)	48 (41.7)	1.7 (1.1–2.6)	
Age, years^c^	55 (34–69)	52 (28–64)	69 (55–78)	1.1 (1.0–1.1)	1·0 (1·0–1·1)	53 (29–64)	49 (22–63)	59 (49–68)	1.0 (1.0–1.0)	1.0 (1.0–1.1)
Female^a^	279 (50.4)	201 (72.3)	78 (28.1)	Ref		184 (48.5)	68 (37.0)	116 (63.0)	Ref	
Male^a^	274 (49.6)	219 (79.9)	55 (20.1)	0.7 (0.4–0.9)		195 (51.5)	67 (34.4)	128 (65.6)	0.9 (0.6–1.4)	
Impaired consciousness^d^	347 (62.6)	244 (70.5)	103 (29.7)	2.5 (1.6–3.9)	2·6 (1·4–4·6)	219 (57.8)	99 (45.2)	120 (54.8)	2.8 (1.8–4.5)	2.1 (1.1–3.7)
Comorbidity^a,e^	182 (35.2)	126 (69.6)	56 (30.8)	1.6 (1.1–2.5)	1·7 (1·0–2·7)	124 (32.7)	55 (44.4)	69 (55.7)	1.7 (1.1–2.7)	1.7 (1.0–2.8)
Steroid therapy^a^	232 (43.3)	191 (82.7)	41 (17.7)	0.5 (0.4–0.8)		170 (46.0)	42 (24.7)	128 (75.3)	0.4 (0.3–0.7)	0.4 (0.3–0.8)
Infecting pathogen^f^										
*N. meningitidis* ^a^	110 (20.0)	103 (93.6)	7 (6.4)	Ref		82 (21.6)	67 (81.7)	15 (18.3)	Ref	
*S. pneumoniae*^*a*^	295 (53.5)	221 (74.9)	74 (25.1)	4.9 (2.2–11.1)		203 (53.56)	116 (57.1)	87 (42.9)	3.4 (1.8–6.3)	
*S. aureus* ^a^	26 (4.7)	10 (38.5)	16 (61.5)	23.5 (7.8–70.7)		10 (2.6)	3 (30.0)	7 (70.0)	10.4 (2.4–45.0)	
*H. influenzae* ^a^	20 (3.6)	18 (90.00)	2 (10.00)	1.6 (0.3–8.5)		17 (4.5)	16 (94.1)	1 (5.9)	0.3 (0.0–2.3)	
Others ^a,h^	77 (14.0)	49 (63.6)	28 (36.4)	8.4 (3.4–20.6)		48 (12.7)	31 (64.6)	17 (35.4)	2.4 (1.1–5.5)	
Other gram negative ^a,g^	23 (4.2)	19 (82.6)	4 (17.4)	3.1 (0.8–11.6)		19 (5.0)	11 (57.9)	8 (42.1)	3.2 (1.1–9.5)	

^a^Number (%).

^b^Missing data in 37 patients.

^**c**^Median (interquartile range).

^d^Impaired consciousness at hospital presentation.

^e^Comorbidities include diabetes, cancer, alcoholism, kidney failure and HIV.

^f^Missing data in 107.

^g^Includes *E· coli, K. pneumonia, P. multocida, Capnocytophaga spp*.

^h^Includes *Streptococcus *spp*, E. faecalis, L. monocytogenes.*

It was possible to examine the contribution of microbial pathogens on death due to CABM in univariate analysis. *S. pneumoniae, S. aureus*, and the ‘other Gram-negative group’ were all associated with a marked increase in the odds of death compared to CABM due to *N. meningitidis* (Table 3).

Multivariable analysis (excluding the data from Dr Miner et al. as these consisted only of patient age, time to antibiotics and outcomes) showed that each hour delay in time to antibiotics was associated with a 10% increase in odds of mortality (adjusted OR 1.1, 95% CI 1.0–1.1). The adjusted analysis indicated that death was associated with advancing age, reduced conscious state at the time of hospital presentation, and the presence of comorbidities. None of the microbial pathogens was significantly associated with death in the adjusted analysis (Table 4).

**Table 4:** Factors associated with mortality and neurological impairment due to community-acquired bacterial meningitis, including time to antibiotics using patients matched for age and sex by propensity scoring.

Covariates	< / > 1 h^a^		< / > 2 h		< / > 3 h		< / > 4 h		< / > 5 h	
	Univariate	Multivariable	Univariate	Multivariable	Univariate	Multivariable	Univariate	Multivariable	Univariate	Multivariable
**Mortality**	n = 226		n = 362		n = 362		n = 324		n = 286	
Time to antibiotics	1.64 (0.81–3.23)	1.02 (1.02–1.02)	2.43 (1.43–4.12)	2.29 (1.28–4.09)	2.93 (1.67–5.12)	2.78 (1.49–6.16)	3.07 (1.81–5.20)	3.77 (2.49–5.71)	2.65 (1.54–4.54)	3.11 (1.47–6.59)
Age (years)	1.06 (1.03–1.08)	1.05 (1.03–1.07)	1.05 (1.03–1.07)	1.05 (1.04–1.05)	1.05 (1.03–1.07)	1.05 (1.04–1.06)	1.06 (1.03–1.08)	1.06 (1.04–1.07)	1.05 (1.03–1.08)	1.05 (1.05–1.06)
Comorbidity	1.30 (0.64–2.64)		1.92 (1.15–3.19)	1.73 (1.24–2.42)	1.61 (0.99–2.60)	1.37 (1.12–1.66)	1.69 (1.01–2.75)		1.46 (0.87–2.46)	
Impaired consciousness^b^	3.83 (1.58–9.27)	3.12 (1.85–5.28)	3.76 (1.98–7.15)	2.95 (2.34–3.73)	2.45 (1.41–4.26)	2.37 (1.89–2.96)	2.34 (1.30–4.18)	2.66 (2.21–3.21)	2.15 (1.19–3.86)	2.37 (1.94–2.90)
Steroid administration	0.60 (0.03–1.27)		0.65 (0.37–1.15)	0.66 (0.55–0.80)	0.60 (0.36–0.99)	0.65 (0.52–0.81)	0.60 (0.35–1.03)	0.64 (0.51–0.79)	0.50 (0.27–0.89)	0.54 (0.40–0.73)
**Neurological impairment**	n = 180		n = 276		n = 263		n = 229		n = 200	
Time to antibiotics	1.62 (0.84–3.14)	1.76 (0.57–3.56)	1.49 (0.87–2.54)	1.45 (0.83–2.53)	1.60 (0.94–2.72)	1.79 (1.03–3.14)	1.44 (0.83–2.51)	1.62 (0.92–2.87)	1.69 (0.94–3.06)	1.87 (1.02–3.44)
Age (years)	1.05 (1.02–1.07)	1.05 (1.03–1.07)	1.03 (1.01–1.05)	1.03 (1.01–1.05)	1.02 (1.00–1.03)		1.01 (0.99–1.03)		1.00 (0.99–1.03)	
Comorbidity	1.30 (0.64–2.64)		1.71 (1.00–2.92)		1.74 (1.02–2.99)		1.76 (1.00–3.11)		1.40 (0.75–2.49)	
Impaired consciousness	3.83 (1.58–9.28)		1.75 (0.98–3.11)		2.25 (1.25–4.04)	2.79 (1.51–5.18)	1.99 (1.10–3.61)	2.18 (1.19–4.00)	1.92 (1.02–3.62)	2.12 (1.11–4.08)
Steroid administration	0.56 (0.27–1.14)		0.46 (0.27–0.81)	0.47 (0.26–0.83)	0.51 (0.30–0.89)	0.46 (0.26–0.52)	0.61 (0.34–1.07)		0.50 (0.27–0.92)	

^a^Less than or greater than hourly interval to the administration of effective antibacterial therapy.

^b^Impaired consciousness at presentation to hospital.

Using the individual patient data available, multivariable analysis showed an uncertain impact of TTA on neurological impairment (aOR 1.0, 95% CI 1.0–1.1) (Table 3). The presence of impaired consciousness at admission and comorbidities remained significantly associated with the likelihood of neurological impairment and, in contrast to the likelihood of death in this analysis, the use of steroids was protective (aOR 0.4, 95% CI 0.3–0.8).

We analysed the relationship of TTA on death and neurological impairment in groups of patients with CABM who were propensity-matched for age and gender. The propensity-matched groups were used to analyse the CABM outcomes in relation to the same discrete hourly intervals to antibiotic administration (less than or greater than one hour up to less than or greater than five hours) as above. The sample sizes for the propensity-matched groups ranged from 180 to 362 patients. Matching for other predictive variables reduced the sample size to the extent that meaningful comparisons were not possible. This analysis gave subtly different results to the above multivariable analysis on the pooled, individual patient data.

**Figure 3:**
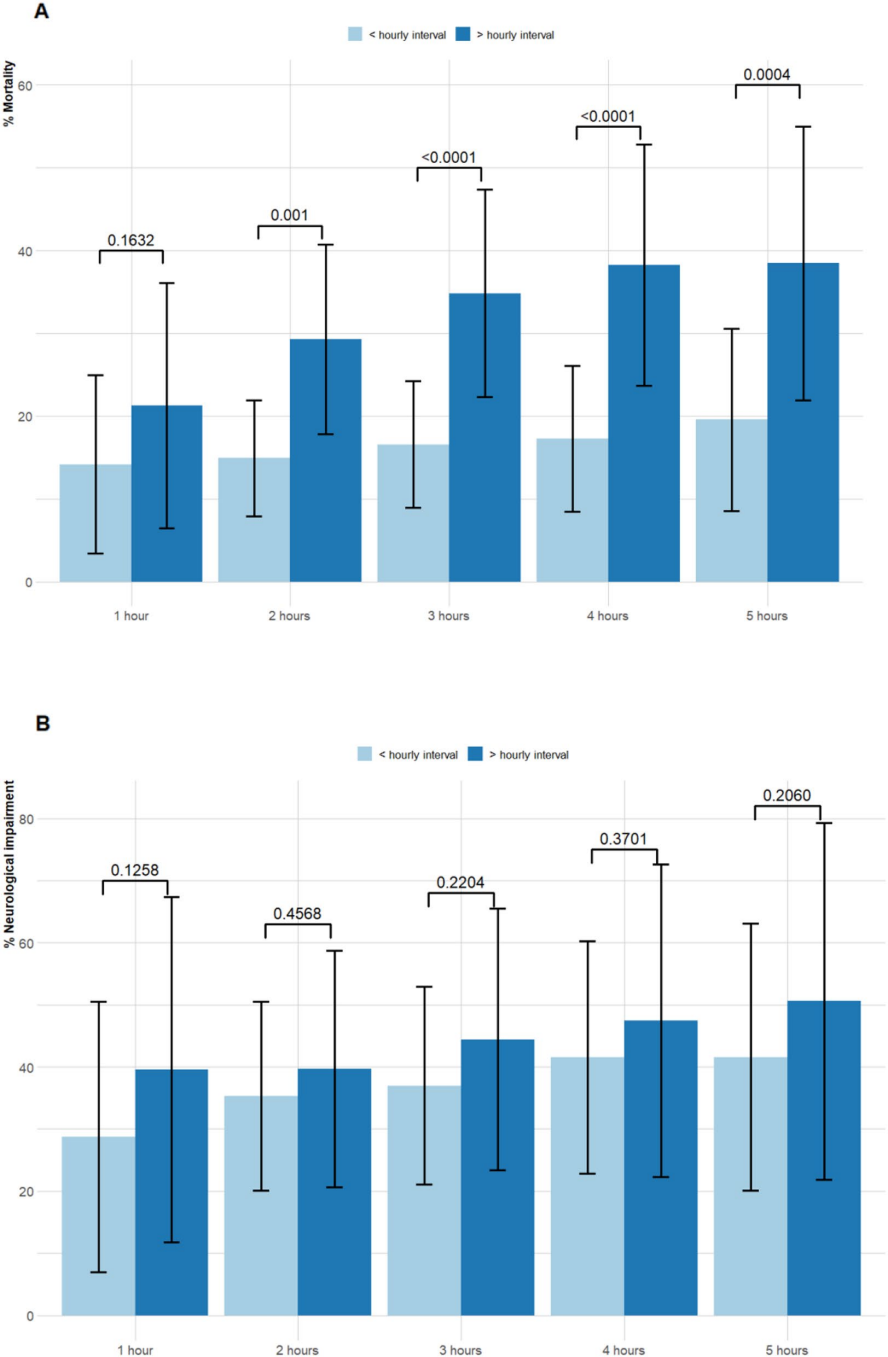
Comparison of (**A**) mortality, (**B**) neurological impairment in relation to time to antibiotics therapy for community-acquired bacterial meningitis in patients matched for age and sex by propensity scoring. (***p < 0.01 Chi-square).

A progressive increase in mortality was shown in the propensity-matched groups that was significantly greater for patients treated after each hourly interval (Table 4 and Fig. 3). Notably, while there was a 2% (aOR 1.02, 95% CI 1.02–1.02) increase in odds of mortality if antibiotic therapy was instituted after one hour, this increased to 129% (aOR 2.29, 95% CI 1.28–4.09) where antibiotics were commenced after two hours. The significant association between age, presence of comorbidities, and decreased conscious state at presentation with increased probability of death were of the same magnitude across the periods of one to five hours to anti-bacterial therapy. The use of steroids had a significant protective effect on mortality that was present after a comparison of less than or greater than two hours to antibiotic therapy. After an interval of three hours, time to antibiotics was significantly associated with the probability of neurological impairment (aOR 1.79, 95% CI 1.03–3.14). As with TTA and CABM mortality the presence of impaired consciousness and steroid administration was also significantly associated with the likelihood of neurological impairment (Table 4 and Fig. 3).

## Discussion

With the use of multivariable analysis of propensity-matched patients, we have shown that progressive delay between presentation to hospital with microbiologically proven bacterial meningitis and treatment with anti-bacterial therapy is associated with increased risk of both death and neurological impairment. After a two-hour delay to antibiotic therapy, the odds of death due to CABM is increased 1.3 fold and continues to rise thereafter. Patients treated before or after one hour had almost the same likelihood of death with an adjusted odds ratio of 1.02. We believe that the very narrow confidence intervals for this and other results are related to precision of the estimated effect size. As such, they are credible even though the lower or the upper limit might be equivalent to the effect size. A similar association was present for the probability of neurological impairment measured by the Glasgow Outcome scale on hospital discharge. And for both adverse outcomes, reduced conscious state at hospital presentation, along with the protective effect of steroid administration, were also significant predictive factors.

These results relied on individual patient data from five cohorts of adult cases of CABM. These include new data on adult patients with CABM treated at TUH along with information from Denmark, UK, and North American hospitals. Our attempt to combine all the aggregate data reported in the published literature on this topic has been thwarted due to the marked heterogeneity of study methodologies with incompatible reporting of TTA. The use of individual patient data allowed for improved precision of our estimates of the association between TTA and death and neurological impairment compared to single-institution studies that have been published previously.

While CABM occurring in children has decreased in incidence due to the use of conjugate vaccines against *S. pneumoniae* and *N. meningitidis*, the burden of disease in adults is increasing. Over the period of 1998–2007 in the USA, the median age of cases of CABM increased from 31 to 42 years and the case fatality rate (~ 16%) remained unaltered^28^. Early initiation of effective anti-bacterial therapy is crucial to the outcome of CABM as the disease progresses rapidly with death and neurological impairment developing as its devastating consequences^2^. The pathogenesis of CABM is complex but death and prolonged neurological impairment result from the severe inflammatory cascade initiated by the virulent causative pathogens^29^. The multiple causes of death include neurological complications such as intracranial bleeding, stroke, status epilepticus, and uncontrolled sepsis^29,30^. Cerebellar tonsillar herniation due to cerebral oedema accounts for 10% of deaths due to CABM^30^. Cerebral arteriopathy due to vasculitis, vasospasm, or intra-arterial thrombosis is the main cause of ischaemia and infarction^31^.

One important caveat associated with this analysis is that overall rates of mortality and neurological impairment are high in comparison to other cohort studies of Western patients^28^. The distribution of causative pathogens from the Danish studies is somewhat atypical in that a significant proportion of cases of adult CABM were due to gram-negative bacteria other than *N. meningitidis* and *H. influenzae,* such as Enterobacteriaceae, *P. multocida,* and Capnocytophaga species. The two Danish studies describe large, community-based samples and they report a very similar distribution of CABM pathogens. CABM due to *E. coli* is rare and has an extremely high mortality rate^32^. *P. multocida*^33^ and *C. canimorsis* are also uncommon causes of meningitis occurring after cat and dog contact^34^. *P. multocida* meningitis has a high mortality while that due to *C. canimorsis* is low but is a frequent cause of long-term neurological impairment. The other studies included here mainly describe patients with the characteristic CABM pathogens, *S. pneumoniae* and *N. meningitidis*.

The main limitation of the study is that while a large number of patient data are available for analysis in this study, they still represent less than a quarter of all the cases described in the literature with a time function related to the commencement of antibiotics. Despite strenuous efforts, it was not possible to obtain other patient data for inclusion. Crucially, due to the observational nature of our data, important confounders may contribute to the measured association between TTA and mortality. For example, earlier antibiotic therapy may be given to patients with more clinically obvious features of CABM and this may correlate with factors associated with good outcomes such as immunological function and lack of frailty, cognitive impairment, or communication difficulty. Indeed, a 2006 systematic review of antibiotic therapy^35^ concluded that confounding of observational study data on meningococcal disease obscured the presence of any benefit from early antibiotic administration.

A recent, randomised trial of early antibiotic therapy administered in ambulances for the broader group of infections causing sepsis failed to show reduced mortality compared with routine practice of treatment commencement in emergency departments^36^. The majority of patients studied had severe sepsis due to pneumonia and < 1% (11/2672) of subjects had central nervous system infection so its direct relevance to the treatment of CABM is limited.

Trials of adjuvant corticosteroids for the treatment of adult CABM show reduced mortality is confined to disease due to *S. pneumoniae*^37^. While these studies do not generally include a time from hospital presentation to antibiotic treatment function it may be that reanalysis of the randomised controlled trial data could provide some additional clarity on the risk of death due to CABM after delayed anti-bacterial therapy.

This study of a large cohort of patients with bacteriologically proven CABM using data from four countries shows that delay of effective treatment for more than two hours is associated with a more than double odds of death. These data could be used to reinforce the appropriate time to antibiotic administration in guidelines for treatment of CABM. Antibiotics must be commenced urgently in patients presenting to our hospitals with clinical features compatible with CABM. This study provides evidence that the time from door to needle must not be more than 2 h.

## Supplementary Information

Supplementary Information.
