# Supplementary material for: Longer than 2 hours to antibiotics is associated with doubling of mortality in a multinational community-acquired bacterial meningitis cohort
Source: Sci Rep. 2022 Jan 13;12:672. doi: 10.1038/s41598-021-04349-7 (PMC8758708; doi:10.1038/s41598-021-04349-7)
Supplement: Supplementary file 1 — Supplementary Information. [file 41598_2021_4349_MOESM1_ESM.docx]

**Supplementary Material to**

Longer than two hours to antibiotics is associated with doubling of mortality in a multinational community acquired bacterial meningitis cohort

Damon P. Eisen^1,2^, Elizabeth Hamilton^1^, Jacob Bodilsen^3^, Rasmus Køster-Rasmussen^4^, Alexander J. Stockdale^5^, James Miner^6^, Henrik Nielsen^3^, Olga Dzupova ^7^, Varun Sethi ^1^, Rachel K. Copson ^1^, Miriam Harings ^1^, Oyelola Adegboye ^8,9*^

1. The Townsville University Hospital, Angus Smith Drive, Douglas, Queensland, Australia, 4814
2. College of Medicine and Dentistry, Discovery Drive, James Cook University, Douglas, Queensland 4814, Australia
3. Department of Infectious Diseases, Aalborg University Hospital, Mølleparkvej 4, 9000 Aalborg, Denmark.
4. The Research Unit for General Practice, Department of Public Health, University of Copenhagen, 1014 Copenhagen K, Denmark
5. Institute of Infection and Global Health, University of Liverpool, Liverpool, L69 7BE, United Kingdom
6. Hennepin County Medical Center, University of Minnesota, Minneapolis, USA
7. Third Faculty of Medicine, Charles University, and University Hospital bULOVKA, Prague, Czech Republic
8. Public Health and Tropica Medicine, College of Public Health, Medical and Veterinary Sciences, James Cook University, Douglas, Queensland 4814, Australia
9. Australian Institute of Tropical Health and Medicine, Discovery Drive, James Cook University, Douglas, Queensland 4814, Australia

*Corresponding author: Oyelola Adegboye

Contact details: Public Health and Tropica Medicine, College of Public Health, Medical and Veterinary Sciences, James Cook University, 1 James Cook Drive, Douglas, Queensland 4814, Australia

Tel: +61 7 47815707

Email: [oyelola.adegboye@jcu.edu.au](mailto:damon.eisen@jcu.edu.au)

Figure S1: PRISMA flow diagram of study search for literature reporting effect of time to antibiotics on outcomes in community-acquired bacterial meningitis

Records screened
(n = 753)

Records after duplicates removed
(n = 753)

Additional records identified through other sources
(n = 3)

## Identification

## Screening

Records identified through database searching
(n = 775)

## Eligibility

## Included

Studies included in qualitative synthesis
(n = 18)

Full-text articles excluded, with reasons
(n = 78)

No time function (n=30)

Full text not traceable (n=8)

No original data available (n=9)

Only children (n=9)

No culture proven meningitis (n=3)

Focus on corticosteroids only (n=3)

Viral or TB meningitis (n=1)

Hospital acquired meningitis (n=1)

Duplicates (n=14)

Full-text articles assessed for eligibility
(n = 96)

Records excluded on the basis of title/abstract
(n = 657)

**Supplementary Table S1: Search strategy for Medline**

| **Search number** | **Search Term** |
| --- | --- |
| 1 | exp Meningitis, Bacterial/ |
| 2 | Bacterial meningitis mp. [mp= title, abstract, original title, name of substance word, subject heading word, keyword heading word, protocol supplementary concept word, rare disease supplementary concept word, unique identifier, synonyms] |
| 3 | 1 or 2 |
| 4 | exp Anti-Bacterial Agents/ |
| 5 | antibiotics. mp |
| 6 | 4 or 5 |
| 7 | exp Time Factors/ |
| 8 | time mp. [mp= title, abstract, original title, name of substance word, subject heading word, keyword heading word, protocol supplementary concept word, rare disease supplementary concept word, unique identifier, synonyms] |
| 9 | 7 or 8 |
| 10 | 3 and 6 and 9 |
| 11 | limit 10 to humans |

**Supplementary Table S2.** International Classification of Disease Edition 10 Australian Modification (ICD-10-AM) discharge codes for bacterial meningitis

A32.1 Listerial meningitis and meningoencephalitis

A39.0 Meningococcal meningitis

G00.0 Haemophilus meningitis

G00.1 Pneumococcal meningitis

G00.2 Streptococcal meningitis

G00.3 Staphylococcal meningitis

G00.8 Other bacterial meningitis

G00.9 Bacterial meningitis unspecified

G01 Meningitis in bacterial diseases classified elsewhere
